# TAF4 Inactivation Reveals the 3 Dimensional Growth Promoting Activities of Collagen 6A3

**DOI:** 10.1371/journal.pone.0087365

**Published:** 2014-02-03

**Authors:** Igor Martianov, Emilie Cler, Isabelle Duluc, Serge Vicaire, Muriel Philipps, Jean-Noel Freund, Irwin Davidson

**Affiliations:** 1 Department of Functional Genomics and Cancer, Institut de Génétique et de Biologie Moléculaire et Cellulaire. CNRS/INSERM/UDS. Illkirch, France; 2 Inserm U1113 - Développement et Physiopathologie de l'Intestin et du Pancréas. Strasbourg, France; Università degli Studi di Milano, Italy

## Abstract

Collagen 6A3 (*Col6a3*), a component of extracellular matrix, is often up-regulated in tumours and is believed to play a pro-oncogenic role. However the mechanisms of its tumorigenic activity are poorly understood. We show here that *Col6a3* is highly expressed in densely growing mouse embryonic fibroblasts (MEFs). In MEFs where the TAF4 subunit of general transcription factor IID (TFIID) has been inactivated, elevated *Col6a3* expression prevents contact inhibition promoting their 3 dimensional growth as foci and fibrospheres. Analyses of gene expression in densely growing *Taf4^−/−^* MEFs revealed repression of the Hippo pathway and activation of Wnt signalling. The Hippo activator Kibra/Wwc1 is repressed under dense conditions in *Taf4^−/−^* MEFs, leading to nuclear accumulation of the proliferation factor YAP1 in the cells forming 3D foci. At the same time, *Wnt9a* is activated and the *Sfrp2* antagonist of Wnt signalling is repressed. Surprisingly, treatment of *Taf4^−/−^* MEFs with all-trans retinoic acid (ATRA) restores contact inhibition suppressing 3D growth. ATRA represses *Col6a3* expression independently of TAF4 expression and *Col6a3* silencing is sufficient to restore contact inhibition in *Taf4^−/−^* MEFs and to suppress 3D growth by reactivating Kibra expression to induce Hippo signalling and by inducing *Sfrp2* expression to antagonize Wnt signalling. All together, these results reveal a critical role for *Col6a3* in regulating both Hippo and Wnt signalling to promote 3D growth, and show that the TFIID subunit TAF4 is essential to restrain the growth promoting properties of *Col6a3*. Our data provide new insight into the role of extra cellular matrix components in regulating cell growth.

## Introduction

TAF4 is a subunit of the general transcription factor TFIID. In vertebrates, the TAF4 family comprises a ubiquitously expressed TAF4 protein and a tissue specific paralogue, TAF4b, required for testis and ovary function [1], [2]. To address the function of mammalian TAF4, we previously inactivated TAF4 in the adult mouse epidermis where its loss results in enhanced EGF signalling and increased keratinocyte proliferation [3]. Inactivation of TAF4 also leads to malignant transformation of chemically induced papillomas and the appearance of invasive melanocytic tumours. Thus, TAF4 acts as a tumour suppressor in the epidermis. We also have generated *Taf4^lox/−^* and *Taf4^−/−^* mouse embryonic fibroblasts (MEFs). In *Taf4^lox/−^* MEFs the TFIID contains predominantly TAF4, whereas in *Taf4^−/−^* MEFs, TAF4b replaces TAF4 to maintain TFIID integrity and cell viability [4]. TAF4-containing and TAF4b-containing TFIIDs have different properties as *Taf4^−/−^* MEFs display TGFβ-dependent autocrine growth and deregulated expression of more than 1000 genes.

Contact inhibition is a process that arrests cell proliferation upon cellular contacts under conditions of high density. It is an important mechanism of anti-cancer defence, as tumour cells normally lose this property and grow in an uncontrolled manner. The molecular mechanisms underlying contact inhibition are still poorly understood. A number of recent studies identified the Hippo signalling pathway as a major effector of contact inhibition [5]–[7]. Activation of the Hippo pathway leads to phosphorylation of the YAP1 and WWTR1/TAZ coactivators by the LATS1/2 kinases and their export from the nucleus. When Hippo signalling is attenuated, YAP and TAZ accumulate in the nucleus acting as coactivators for various transcription factors, such as those of the TEAD family, that activate genes promoting cell proliferation [8]. In normal cells, Hippo signalling is activated in dense conditions leading to export of YAP/TAZ from the nucleus and arrest of proliferation, while in transformed cells lacking contact inhibition the Hippo signalling pathway is attenuated and YAP/TAZ remain in the nucleus even under dense conditions to promote cell growth.

Loss of contact inhibition is not the only event in oncogenic transformation. Other events are required, such as the activation of the Wnt signalling pathway. This pathway regulates many biological processes, including morphology, proliferation, motility and cell fate. The canonical Wnt pathway involves binding of Wnt proteins to cell-surface receptors of the Frizzled family, causing the receptors to activate Dishevelled family proteins and resulting in stabilization and nuclear import of β-catenin. Inappropriate activation of this pathway with accumulation of nuclear β-catenin is observed in several human cancers [9].

It is becoming increasingly recognised that the extracellular matrix (ECM) not only provides a 3 dimensional (3D) matrix for cell growth and organogenesis, but that signals from the ECM play critical roles in cell fate and cell growth [10]. In cancer, the local microenvironment and especially the ECM also play an important role in cancer progression. Collagens are the most abundant proteins in the ECM and Collagen VI has been the focus of substantial interest due to its association with cancer. Collagen VI is a large, multidomain ECM protein composed of a triple-helix of α1, α2, and α3 chains that tetramerise through end-to-end association and assemble into a microfibrillar network. It was shown that Collagen VI is up-regulated during murine mammary tumour progression [11]. Accordingly, the absence of Collagen VI in a breast cancer-prone mouse strain reduced the rates of early hyperplasia and primary tumour growth [12]. Similarly, Collagen VI has been shown to contribute to the resistance of human ovarian cancer cells to cisplatin treatment and to be up-regulated in several high grade human tumours [13]. However, despite its importance, the pathways linking Collagen VI to carcinogenesis remain poor characterised. Moreover, Collagen VI is also a component of the ECM in normal tissue, indicating that other mechanisms may keep in check its oncogenic activity.

Here we show that TAF4 is able to attenuate the growth promoting activities of Collagen VI. In a *Taf4^−/−^* background, a subpopulation of MEFs loses contact inhibition, resulting in the formation of 3D foci and growth as fibrospheres. The cells forming foci are characterised by activated Wnt signalling and inhibition of Hippo signalling. Strikingly, Col6a3 silencing is sufficient to restore contact inhibition in *Taf4^−/−^* MEFs. Our data show for the first time that *Col6a3* plays a role in modulating signalling pathways involved in contact inhibition providing an explanation for the observed association between Col6a3 and cancer. It also suggests that changes in the ratio of TAF4 and TAF4b can play a role in the susceptibility of cells to Col6a3-promoted 3D growth. Finally, we also show that all-trans retinoic acid (ATRA) treatment represses Col6a3 expression thus abrogating premalignant changes in both wild-type and *Taf4^−/−^* MEFs. Our data suggest that ATRA could be a valuable treatment for refractory Collagen VI-associated cancers.

## Materials and Methods

### Cell lines

The C1*Taf4* ^lox/−^ and C3*Taf4* ^−/−^ MEFs were derived from genetically modified *Taf4* ^lox/−^ mouse embryos and have previously been described [4]. The floxed *Taf4* allele was defloxed in the C1 MEFs by expression of the Cre recombinase and loss of TAF4 expression in the C3 MEFs was verified by PCR genotyping and by western blot analysis as described (10). Cells were cultured in Dulbecco's minimal essential media (DMEM) supplemented with 4.5 g/l glucose and 10% foetal calf serum. Cells were treated with 10^−6^ M ATRA dissolved in ethanol (DMSO) as indicated.

### Fibrosphere assays

For fibrosphere assays, the indicated cells were also grown under non-adherent conditions in bacterial culture plates. 10^6^ of the indicated cells were inoculated and grown for 10 days.

### Immunofluoresence

Immunofluoressence on C1 and C3 cells was performed by standard procedure using following antibodies: COL6A3 (PAB17517, Abnova), CTNNB1 (Abcam, ab6302, and BD Biosciences, 610153), YAP1 (Cell Signalling, #4912), TAZ (BD Bionsciences, 560235), SOX2 (Cell Signalling, #4900). Immunofluorescence was visualized using a Zeiss Axiophot (Carl Zeiss, Gottingen, Germany) microscope equipped with epifluorescence illumination. Confocal microscopy was performed on a Leica SP2 microscope.

### Wnt signalling inhibitors

IWR-1 (Sigma-Aldrich, I0161) and XAV939 (Sigma-Aldrich, X3004) were used at 1 µg/mL concentration.

### RNA-seq

RNA-seq was performed as previously described [14]. Briefly, mRNA was purified from 2 µg of total RNA from C3 cells, grown at low or high densities, as spheres or after 12 and 24 hours of treatment with ATRA, using oligo-dT magnetic beads and fragmented using divalent cations at 95°C for 5 minutes. The cleaved mRNA fragments were reverse transcribed to cDNA using random primers and SuperScript II reverse transcriptase (# 18064-014, Invitrogen) and second strand cDNA synthesis using Polymerase I and RNase H. DNA libraries were prepared as indicated by Illumina and checked for quality and quantified using 2100 Bioanalyzer (Agilent, USA). The libraries were loaded in the flowcell at 6pM concentration and clusters were generated using the Cbot and sequenced on the Illumina Genome Analyzer IIx as single-end 54 base reads following Illumina's instructions. Sequence reads mapped to reference genome mm9/NCBI37 using Tophat [15]. Quantification of gene expression was done using Cufflinks [16] and annotations from Ensembl release 57. For each transcript the resulting FPKM were converted into raw read counts and these counts were added for each gene locus. Data normalization was performed as described by Anders et al. [17] and implemented in the DESeq Bioconductor package. For the analysis of gene expression in low vs high-density C3 cells and fibrospheres, cut off values were, a minimum average RPKM value of 5, and fold changes ≥3 or ≤0.33 with a pvalue of ≤0,05. For the analysis of gene expression following retinoic acid treatment, fold changes ≥2 or ≤0.5 were considered along with a pvalue ≤0,05.

### ShRNA-mediated gene silencing

Lentiviral shRNA expression vectors and packaging plasmids were purchased from Sigma-Aldrich. The TRC numbers are indicated in the Supplemental information. Lentiviral particles were generated by co-transfection of shRNA vector together with packaging plasmids into 293T cells. Medium was changed 24 hs after transfection and viruses harvested 48 hs after transfection and used for infection of C3 cells. Infected C3 cells were selected with puromycin resistant and silencing of targeted gene was checked by RT-qPCR.

### RT-qPCR

RNA was prepared using Trizol reagent (Invitrogen, 15596-018) according to manufacturer's protocol. Reverse transcription was performed with Super Script II reverse transcriptase (Invitrogen, 18064-022) as described in manufacture's protocol. Random hexaoligonucleotides were used as primers. qPCR was performed using LightCycler 480 SYBR Green I master mix (Roche, 04887352001) on the LightCycler 480 Real-Time PCR System. List of oligonucleotides is supplied in x S1.

### Proliferation assays

For cell counting, 5×10^4^ cells were seeded in 10 cm plates. At the indicated times the cells were trypsinised and counted. BrdU incorporation was performed using a BrdU Cell Proliferation Assay kit (QIA58) from Merck/CalBiochem as per the manufacturers instructions. Briefly, the cells were grown for the indicated times with or without RA and then BrdU incorporation for 24 hours was measured by an immuno-colometric assay.

## Results

### TAF4-null MEFs display 3D growth

**Figure 1:**
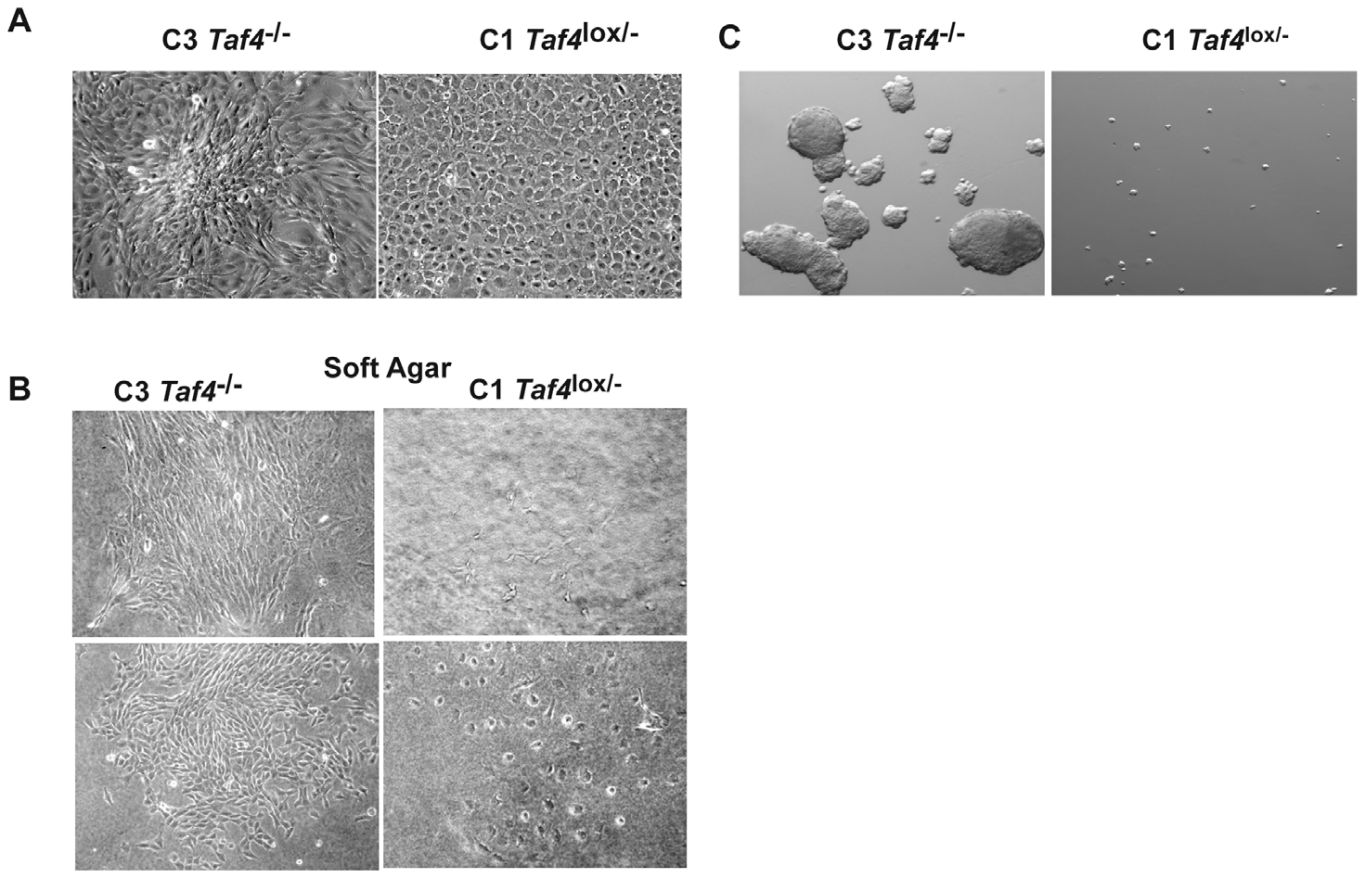
3D growth of C3 cells. **A**. Phase contrast microscopy (20× magnification) of C1 and C3 cells grown as dense cultures for 3 days. **B**. Growth of C1 and C3 cells after 10 days in soft agar. **C**. Phase contrast microscopy (12× magnification) of C1 cells or C3 cells in grown for 10 days under non-adherent conditions.

We have previously reported generation of *Taf4^−/−^* MEFs by defloxing of *Taf4^lox/−^* MEFs [4]. The *Taf4^−/−^* MEFs (hereafter C3) are irregularly shaped and have lost contact inhibition as they readily form three dimensional (3D) foci that are never observed with the *Taf4^lox/−^* MEFs (hereafter C1) (Fig. 1A). We also noted that whereas the C1 cells did not proliferate in soft agar, the C3 cells formed clearly visible colonies (Fig. 1B).

Many types of transformed cells can be grown under non-adherent conditions as spheres such as ‘mammospheres’ in the case of breast cancer cells [18]. We tested the ability of the C3 cells to grow as ‘fibrospheres’ under non-adherent conditions and found that they form large round spheres and trabecular structures, whereas the C1 cells did not develop fibrospheres (Fig. 1C). TAF4 inactivation therefore confers the ability for 3D growth to at least a subpopulation of C3 MEFs.

### Gene expression changes associated with growth at high density and as fibrospheres

**Figure 2:**
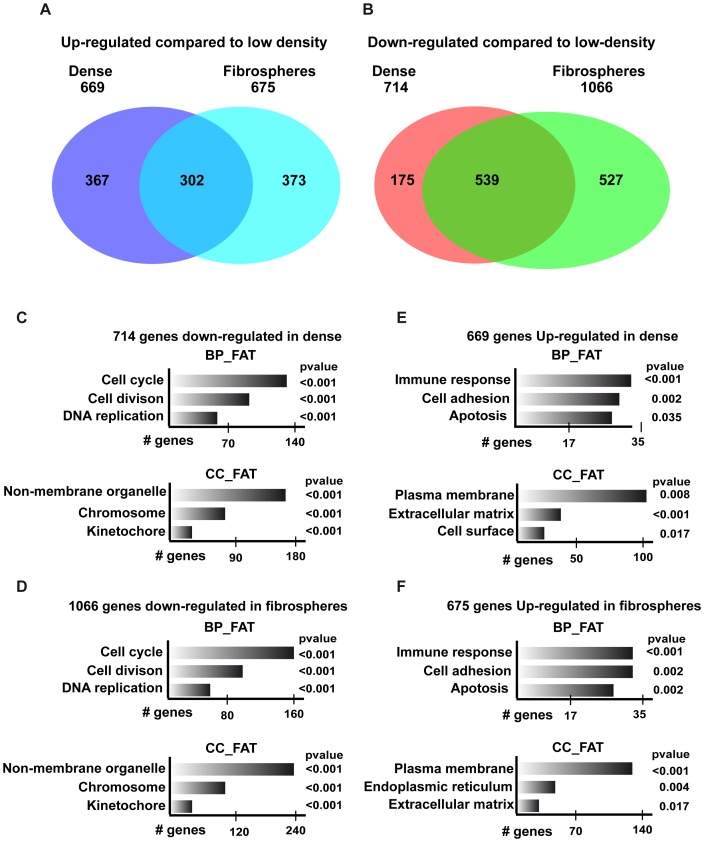
Changes in gene expression upon 3D growth of C3 cells. **A–B** Venn diagrammes showing overlapping changes in gene expression upon growth as dense cultures or fibrospheres. **C–F**. Ontology analysis (http://david.abcc.ncifcrf.gov/) of the deregulated genes showing some relevant categories from the CC-FAT and BP FAT classifications. The number of genes and the associated *P* values for each category are indicated.

We next used RNA-seq to profile the changes in gene expression that occur upon 3D growth. RNA was prepared from low or high-density adherent cultures of C3 cells comprising 3D foci and from fibrospheres. In comparison with non-confluent cells, 669 transcripts were-up-regulated and 714 down-regulated in dense cells (Figs. 2A and B and Table S2). Similarly, in fibrospheres, 675 transcripts were-up-regulated and 1066 down-regulated in comparison with non-confluent cells (Figs. 2A and B and Table S2). A large overlap can be observed amongst transcripts whose expression is deregulated upon the transition from the non-confluent to the dense or fibrosphere states. Around half of the up-regulated genes and a majority of the down-regulated genes are regulated in common under both the dense and fibrosphere conditions.

Ontology analysis of the down-regulated transcripts indicated strong enrichment in those involved in cell cycle and cell division consistent with the fact that proliferation is considerably reduced in dense C3 cells or when grown as spheres compared to the rapid growth of the non-dense cultures (Figs. 2C and D and Table S3). In contrast, the up-regulated transcripts are enriched in three distinct classes, those associated with activation of the interferon (immune) response, apoptosis, and transcripts encoding proteins involved in modification of adhesion and ECM composition (Fig. 2E and F). An ontology analysis of the transcripts that are selectively up-regulated in fibrospheres compared to dense monolayers also revealed an enrichment in genes associated with the membrane and ECM, but did not reveal a pathway specific to this growth state (data not shown).

We had previously reported that genes of the interferon response were strongly induced in the C3 cells lacking TAF4 compared to the C1 cells. We ascribed this difference to TAF4 inactivation [4]. However, the above results indicate that activation of the interferon response requires loss of TAF4 and growth to high density. The presence of a large collection of apoptosis associated transcripts amongst the up-regulated class further indicates the presence of a significant number of apoptotic cells in dense cultures and in fibrospheres. The presence of the apoptotic cells may be related to activation of the interferon response genes, since it has been previously established that DNA from dead cells can induce the interferon response if taken up by the surrounding cells [19]. However, in the context of this study we have not further investigated this point.

**Figure 3:**
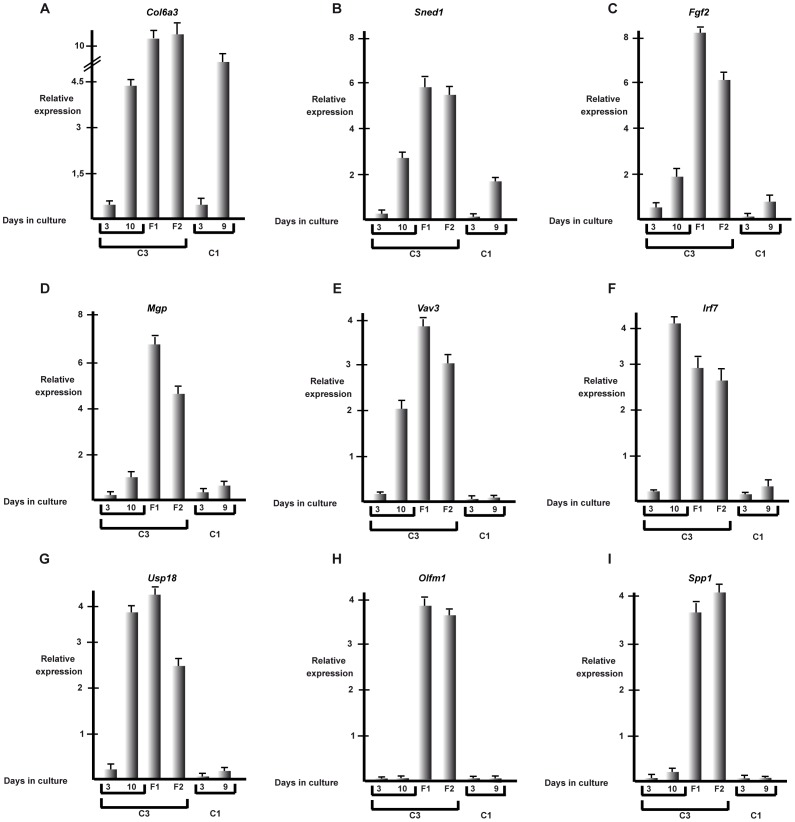
Comparative gene expression in C1 and C3 cells. **A–I**. Results of RT-qPCR analysis of the indicated genes in C1 or C3 cells grown for 3 and 10 days in monolayer cluture or 10 days as fibrospheres (F1 and F2).

The gene expression analysis also clearly indicates major changes in transcripts associated with cell adhesion and the ECM in dense cultures and spheres. Expression of several collagens (6a3, 18a1) and laminins (a4 and a5) are strongly induced along with VCAM1, thrombospondin 2, intergrins b8, a2b and a7 and cadherins 13 and 26. Thus, 3D growth involves major changes in cell adhesion and remodelling of the ECM. On the other hand, expression of *Olfm1* and *Spp1*, although induced in dense C3 cells, is very much higher in fibrospheres (Figs. 3H and I).

We confirmed the changes in expression of several of the genes identified by RNA-seq by RT-qPCR in low density C3 cells and after increasing times in culture to become dense and in fibrospheres. In agreement with the RNA-seq data, expression of *Col6a3* strongly increases in dense C3 cells and is strongly expressed in fibrospheres (Fig. 3A). A similar profile was seen with *Sned1*, *Fgf2*, *Mgp*, *Vav3* and the interferon response genes *Irf7* and *Usp18* (Figs. 3B–G).

**Figure 4:**
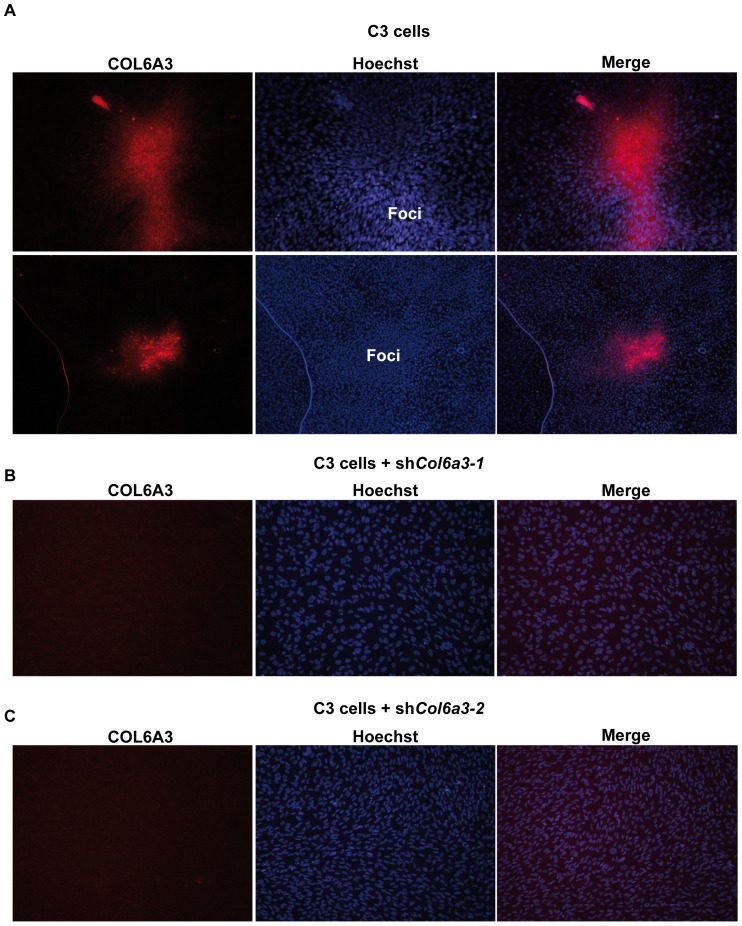
Elevated COL6A3 expression in MEFs growing as 3D foci. **A**. Immunostaining of dense C3 MEFs for COL6A3. Two distinct fields are shown. COL6A3 is not homogeneously expressed, but strong expression is limited to the cells forming the 3D foci. B–C. Immunostaining of dense C3 cells expressing shRNAs directed against *Col6a3* demonstrating the specificity of the staining.

Expression of the interferon response genes was not induced in dense C1 cells and is thus specific to C3 cells. Interestingly however, expression of several of the ECM-related genes such as *Col6a3* and *Sned1* is significantly induced in dense TAF4-expressing C1 cells showing that their up-regulation is more generally associated with dense MEF growth and is not specific to the C3 cells (Figs. 3A–C). Strikingly, immunofluorescence using anti-COL6A3 antibody shows that strong COL6A3 expression in *Taf4^−/−^* MEFs was limited to the cells forming the 3D foci (Fig. 4A). The surrounding monolayer cells show only weak labelling. The immunofluorescence signal was lost in cells expressing shRNAs directed against Col6a3 (see below) demonstrating its specificity (Fig. 4B and C). Thus, COL6A3 expression is strongly and selectively up-regulated in the C3 cells that form 3D foci.

### Suppressed Hippo signalling in C3 MEFs

The Hippo signalling pathway is a major player in contact inhibition where cell-cell contacts activate the pathway to phosphorylate the YAP1 and WWTR1/TAZ coactivators and export them from the nucleus of dense cells thereby leading to arrest of proliferation. Examination of the gene expression profiles of low vs high density C3 cells and fibrospheres shows no significant change in the expression of many of the principal components of this pathway with the notable exception of Kibra (*Wwc1*) whose expression is strongly down regulated in dense cells and fibrospsheres (Table S2). Kibra is an activator of the Hippo pathway in *Drosophila in vivo* [20] In mammalian cells, Kibra associates with and activates the LATS1-2 kinases promoting YAP1 phosphorylation and its nuclear export [21]. In contrast, silencing of Kibra expression reduces YAP1 phosphorylation resulting in nuclear accumulation. We also noted that *Fat4* expression was down-regulated in fibrospheres compared to non-confluent cells, although the expression of this gene is overall low in C3 cells (Table S2). FAT4 is an activator of Hippo signalling in *Drosophila*, but does not appear to regulate the Hippo pathway in mammals [22].

**Figure 5:**
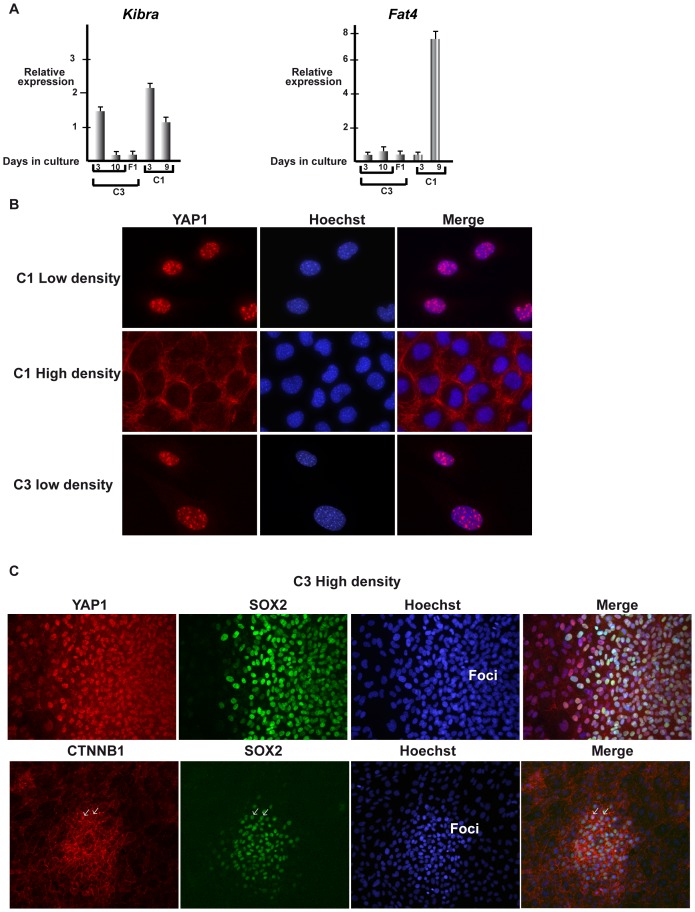
Differential regulation of Hippo signalling in C1 and C3 MEFs. **A** Results of RT-qPCR analysis of the indicated genes in C1 or C3 cells grown for 3 and 10 days in monolayer culture, or 10 days as fibrospheres (F1). **B** Immunostaining of C1 and C3 MEFs for YAP1 at low or high densities. **C** Immunostaining of C3 MEFs grown at high density for YAP1, SOX2 or β-catenin as indicated.

RTqPCR confirmed that Kibra expression is reduced in dense C3 cells and in fibrospheres (Fig. 5A). In contrast, its expression is considerably higher in non-dense and dense C1 cells. These results show that Kibra, a positive regulator of Hippo signalling is strongly expressed in C1 cells, but is down regulated in dense C3 cells suggesting that the Hippo pathway is activated in dense C1 cells leading to contact inhibition, but not in C3 cells. RTqPCR also confirmed low expression of *Fat4* in dense and non-dense C3 cells as well as fibrospheres, whereas its expression is strongly induced in dense C1 cells (Fig. 5A).

To determine whether reduced Kibra expression correlates with Hippo signalling, we assessed cellular localisation of YAP1 and TAZ by immunostaining of C1 and C3 cells. In low density proliferating C1 cells, a clear nuclear staining for YAP1 is observed (Fig. 5B). In contrast, in dense C1 cultures where proliferation is arrested, YAP1 is now predominantly localised in the cytoplasm at the plasma membrane. In proliferating C1 cells, TAZ is also localised in the nucleus, whereas in dense cells, it remains in the nucleus, but the signal becomes weaker and in many cells is almost completely lost (Fig. S1A). These observations are consistent with activation of Hippo signalling under dense conditions that leads both to cytoplasmic localisation of YAP1, but also to proteolytic degradation of TAZ [23].

In C3 cells a different profile is observed. In low-density C3 cells, YAP1 and TAZ are nuclear (Figs. 5B and Fig. S2A). In dense C3 cells, TAZ remains nuclear, but its expression is reduced with many cells showing little or no staining (Fig. S2A). In dense C3 cultures however, YAP1 is located in both the nucleus and cytoplasm in cells that are dense, but not forming 3D foci. In contrast, in cells forming 3D foci, strong YAP1 nuclear staining is observed (Fig. 5C and Fig. S2C). Thus, down-regulation of Kibra in dense C3 cells is associated with a strong increase in YAP1 nuclear localisation accounting for the lack of contact inhibition and the observed 3D foci.

### Wnt signalling is essential for 3D growth of C3 MEFs

**Figure 6:**
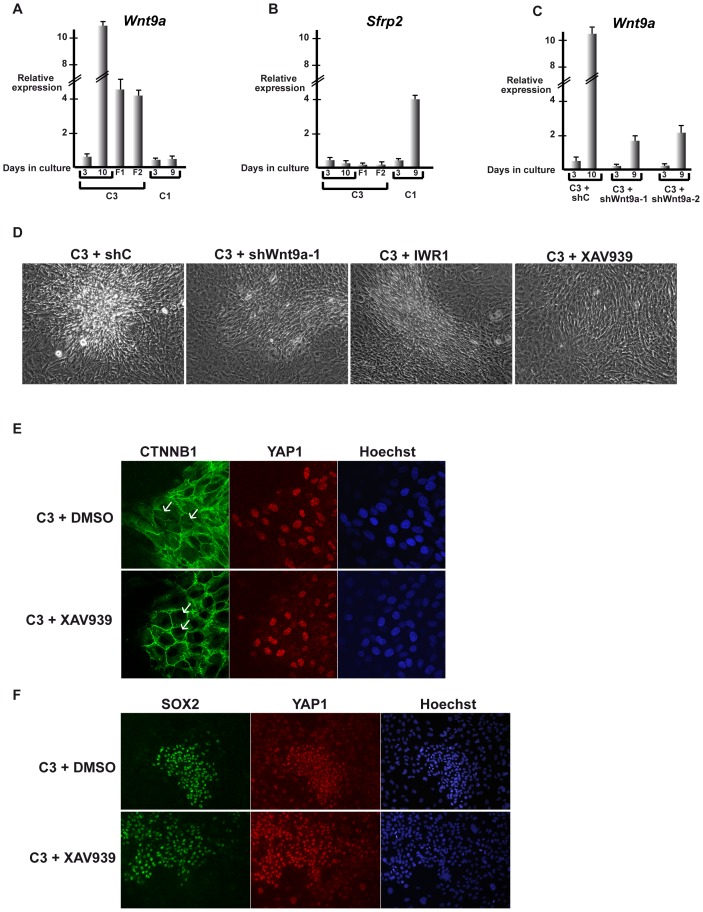
Wnt signalling is required for 3D growth. **A–C**. Results of RT-qPCR analysis of the indicated genes in C1 or C3 cells grown for 3 and 9–10 days in monolayer cluture, 10 days as fibrospheres (F1 and F2) or in C3 cells expressing the indicated shRNAs. **D** Phase contrast microscopy (20× magnification) of C3 cells grown in presence or absence of the indicated Wnt pathway inhibitors or expressing control shRNA and shRNA directed againt *Wnt9a*. **E**. Confocal microscopy sections through foci grown in the presence or absence of XAV939 showing that the nuclear β-catenin localisation seen in the control C3 cells is lost in presence of XAV939. **F**. Immunostaining for YAP1 and SOX2 in foci in presence or absence of XAV939.

To identify additional signalling pathways that may be involved in promoting the 3D growth of C3 cells, we made a closer analysis of the gene expression data. Examination of genes deregulated in densely growing cells showed that expression of the *Wnt9a* ligand was strongly up-regulated, while the Wnt antagonist *Sfrp2* was repressed (Table S2). RT-qPCR confirmed that *Wnt9a* expression is strongly up-regulated in dense C3 cells and also showed its up-regulation in fibrospheres that was not evidently seen by RNA-seq. In contrast, *Wnt9a* is not induced in dense C1 cells (Fig. 6A). Furthermore, basal *Sfrp2* expression in low density C3 cells is further reduced when cells become dense, whereas it is strongly up-regulated in dense C1 cells (Fig. 6B). These data suggest that up-regulation of the *Wnt9a* ligand and the reduction in *Sfrp2* expression activates the Wnt pathway to promote 3D growth of C3 cells, whereas in dense C1 cells Wnt signalling is attenuated due to lack of *Wnt9a* induction and high *Sfrp2* expression.

To test the role of Wnt signalling in 3D growth, we performed shRNA knockdown of *Wnt9a* and tested the effect of chemical inhibitors of this pathway. *Wnt9a* silencing with two independent shRNAs (Fig. 6C) as well as treatment with the Wnt pathway inhibitors IWR1 and XAV939 all reduced 3D foci formation (Fig. 6D and data not shown). In each case, upon prolonged growth, areas of high cell density form, but they do not develop into full 3D foci as seen with untreated cells expressing a control shRNA. Furthermore, each treatment also completely inhibited fibrosphere formation (data not shown). These data show that Wnt signalling plays a critical role in 3D growth of the C3 cells.

Cross-talk between the Hippo and Wnt signalling pathways has been described. Hippo can negatively regulate Wnt signalling, for example, through interactions of cytoplasmic TAZ with the dishevelled proteins [24]. In contrast, in the absence of Hippo signalling, nuclear YAP can associate with β-catenin (CTNNB1) to promote expression of target genes such as SOX2 [25]. Formation of 3D foci is associated with nuclear β-catenin staining as well as nuclear YAP1 accumulation (Figs. 5C and 6E). A low expression of SOX2 can be seen in both low and high-density C1 cells (Fig. S1B). In low density C3 cells, SOX2 expression is heterogeneous, with cells that express little or no SOX2, cells with intermediate levels and rare cells with strong SOX2 staining (Fig. S2B). Under dense conditions, SOX2 is strongly expressed in the cells that form foci (Fig. 5C and Fig. S2B). Almost all cells in foci with strong nuclear YAP1 staining also display strong SOX2 staining. In control experiments and as expected, strong SOX2 staining is seen in all nuclei of F9 embryonal carcinoma cells, but is absent from hepatocyte cells demonstrating the specificity of this staining (Fig. S1C). Treatment with the Wnt inhibitor XAV939 leads to a loss of nuclear β-catenin staining (Fig. 6E), but has no effect on either SOX2 or YAP1 expression or localisation (Fig. 6F). Thus, inhibition of Wnt signalling does not lead to a loss of SOX2 expression suggesting that in these cells nuclear YAP1 alone is sufficient to promote its expression as is seen in ES cells [26].

Together these results show that the conjugation of enhanced Wnt signalling along with reduced Hippo signalling and nuclear YAP1 accumulation is associated with 3D growth of a subpopulation of C3 cells.

### ATRA suppresses 3D growth of *Taf4^−/−^* MEFs

**Figure 7:**
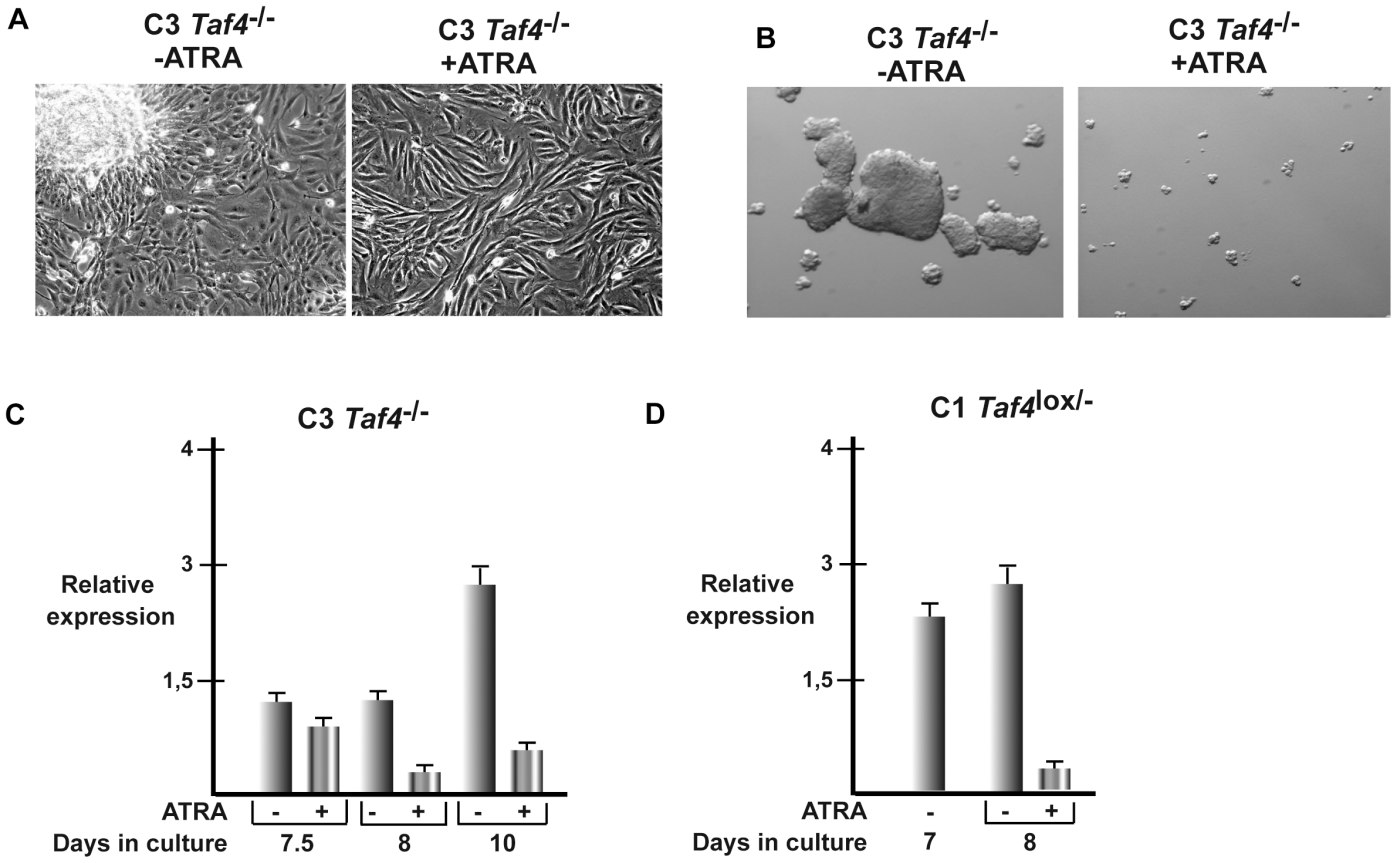
ATRA induced changes in properties of C3 MEFs. **A**. Phase contrast microscopy (20× magnification) of C3 cells grown as dense cultures for 3 days in presence or absence of ATRA. **B**. Phase contrast microscopy (12× magnification) of C3 cells in grown for 10 days as fibrospheres in presence or absence of ATRA. **C**–**D** Effect of ATRA on *Col6a3* expression in C3 and C1 cells grown for the indicated number of days in presence or absence of ATRA.

We have previously shown that ATRA treatment induces a change in morphology of C3 cells that adopt a more regular elongated shape [4]. ATRA treatment did not alter cell proliferation. Growth kinetics performed by cell counting did not show any significant difference between the untreated and ATRA treated C3 cells (Fig. S3A). Similarly, ATRA treatment did not lead to significant changes in BrdU incorporation over a 5 day period (Fig. S3B). Finally ATRA treatment did not modify the cell cycle as measured by FACS analysis (Fig. S3C). In particular, no significant G1/S arrest similar to what is seen with F9 or breast cancer cells [27]–[29] is observed with the MEFs. ATRA does not therefore inhibit 3D growth by inducing cell cycle arrest or apoptosis of the C3 cells. Nevertheless, ATRA restores contact inhibition attested by lack of 3D foci in monolayers (Fig. 7A) and loss of fibrosphere growth (Fig. 7B). These results show that while ATRA does not affect C3 cell growth under normal conditions, it strongly reduces their 3D growth.

### ATRA modulates expression of ECM components

3D growth of C3 cells involves enhanced Wnt signalling and repressed Hippo signalling leading to changes in expression of a large number of genes. We reasoned that expression of one or several of these pathway/genes may be counteracted by ATRA thus inhibiting 3D growth. We used RNA-seq to assay the changes in gene expression after 12 hours of RA treatment to identify mainly direct RAR targets and after 72 hours when the changes in cell morphology become particularly evident to identify transcripts that are induced or repressed under these conditions (Table S4).

After 72 hours of ATRA treatment only 80 genes showed a three-fold or more increase in expression, 32 of which showed also at least a three-fold increase after 12 hours (Table S4). 42 transcripts showed a three-fold or more down-regulation of which 8 were repressed at least three fold after 12 hours (Table S4). Remarkably however, comparison of the genes whose expression is up and down regulated upon 3D growth with those regulated by ATRA reveals Col6a3 gene as almost the only potentially relevant target. Indeed, *Col6a3*, whose expression is strongly induced upon 3D growth, is repressed after 72 hours of ATRA treatment (Table S4). ATRA also down-regulates, but to a lesser extent other collagens *Col6a2*, *Col5a3* and *Col7a1* showing that one of its major effects in these cells is regulation of genes contributing to ECM modification. Repression of *Col6a3* and *Col6a2* expression therefore down regulates assembly of holo-collagen VI potentially modulating the growth and clonogenic properties of C3 cells.

To examine the possible implication of *Col6a3* in 3D cell growth, we first verified by RT-qPCR that its expression was repressed by ATRA. We examined the effect of ATRA on *Col6a3* expression at the time when its expression is induced in dense cells. C3 cells were grown for 7 days in culture until dense, but before the appearance of 3D foci and then exposed to ATRA. After 12 hours of ATRA treatment only a negligible reduction of expression was observed, however after 24 hours a 3–4 fold reduction was observed (Fig. 7C). A more potent reduction in *Col6a3* expression was also seen after 72 hours of treatment when its expression is strongly up-regulated without ATRA upon the formation of 3D foci. Interestingly, ATRA also represses Col6a3 expression in TAF4-expressing C1 cells (Fig. 7D). Hence, ATRA can repress the induction of *Col6a3* expression that takes place upon dense MEF growth, irrespective of the presence or absence of TAF4.

### COL6A3 silencing restores contact inhibition

**Figure 8:**
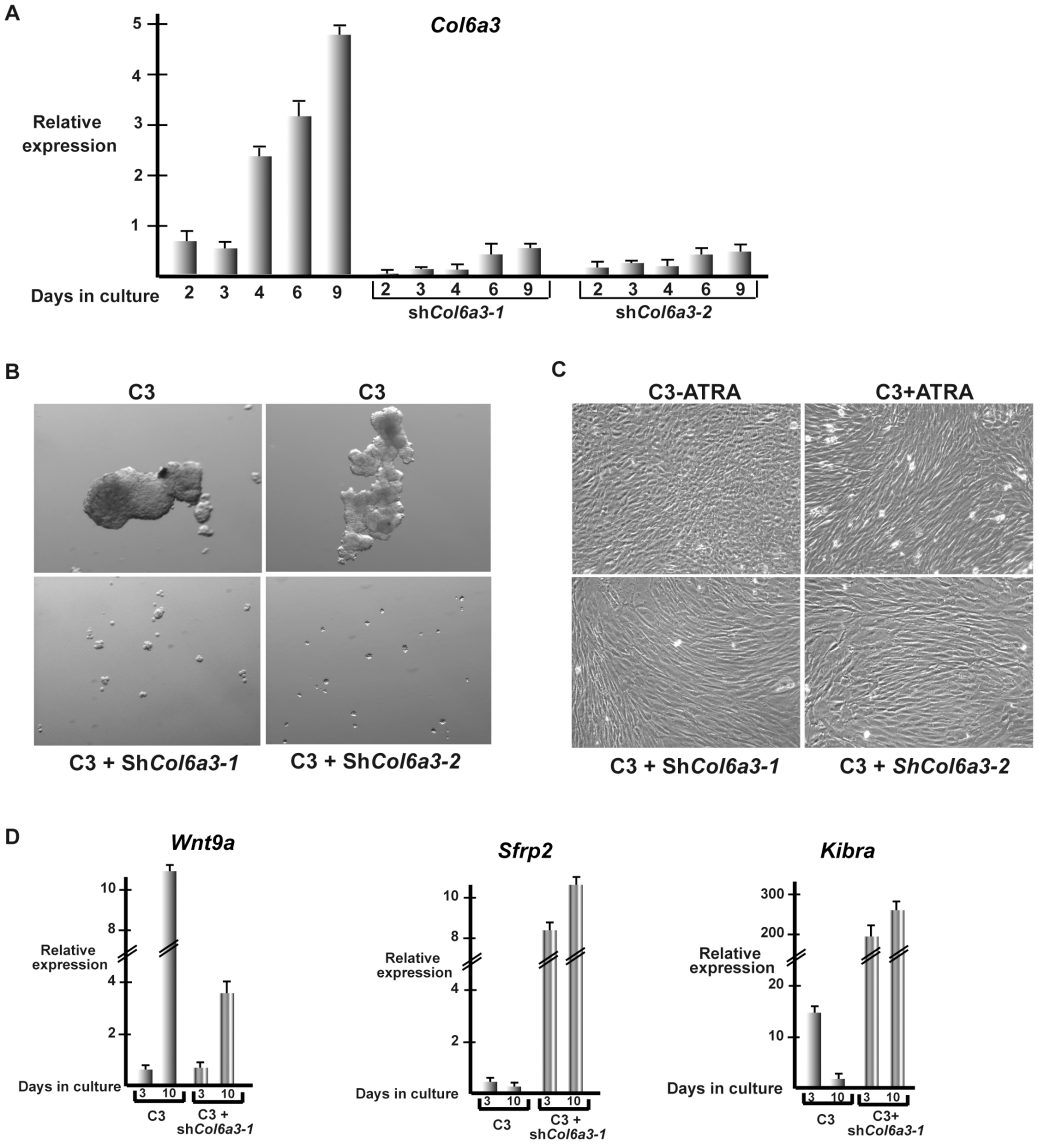
*Col6a3* is required for 3D MEF growth. **A**. RT-qPCR of *Col6a3* expression in cells expressing control shRNA or two independent shRNAs directed against *Col6a3* after the indicated number of days in culture. **B**. Phase contrast microscopy (12× magnification) of C3 cells expressing control shRNA or shRNAs directed against *Col6a3* grown for 10 days as fibrospheres. **C**. Phase contrast microscopy (20× magnification) of C3 MEFs in the presence and absence of ATRA showing the characteristic changes in cell morphology induced by RA and compared with C3 cells expressing shRNAs directed against *Col6a3*. *Col6a3* knockdown induces changes in cell morphology analogous to those seen in the presence of RA. **D**. Effects of *Col6a3* silencing on gene expression.

The above results show a correlation between high expression of COL6A3 in cells forming 3D foci, and the attenuation of this strong expression when foci formation is abolished by ATRA. However, these observations do not demonstrate that elevated COL6A3 expression is essential for 3D growth. To address this point, we used lentiviral shRNA vectors to suppress *Col6a3* expression. Two independent shRNA-sequences showed a significant knockdown, strongly suppressing *Col6a3* expression under high density conditions (Fig. 8A). Strikingly, suppression of *Col6a3* expression by both shRNAs abolished fibrosphere growth (Fig. 8B). Moreover, sh*Col6a3* silencing leads to changes in cell morphology very similar to those seen upon ATRA treatment. Upon knockdown of *Col6a3* expression or ATRA treatment, cells adopt a more regular elongated shape and there was a complete loss of 3D foci formation (Fig. 8C). Strong *Col6a3* expression is therefore essential for formation of 3D foci, but also the general change in morphology seen upon shRNA knockdown shows that even the low basal *Col6a3* expression in monolayer cells plays a role in determining their morphology.

Examination of gene expression in dense sh*Col6a3* knockdown cells showed little effect on many genes, such as *Irf7* and the other interferon response genes or *Sned1*, *Fgf2* or *Mgp*, whose expression was induced upon dense growth of both the control and sh*Col6a3* knockdown (Fig. S4 and data not shown). In contrast, expression of other genes normally induced upon 3D growth such as *Vav3* and *Blnk* is strongly reduced upon *Col6a3* knockdown (Fig. S4). These observations distinguish genes whose expression is -up-regulated in dense cultures of C3 cells and often (with the exception of the interferon response genes) in dense C1 cells that are not dependent on 3D growth, from genes whose expression is associated with 3D growth and are not up-regulated in dense C1 cells.

**Figure 9:**
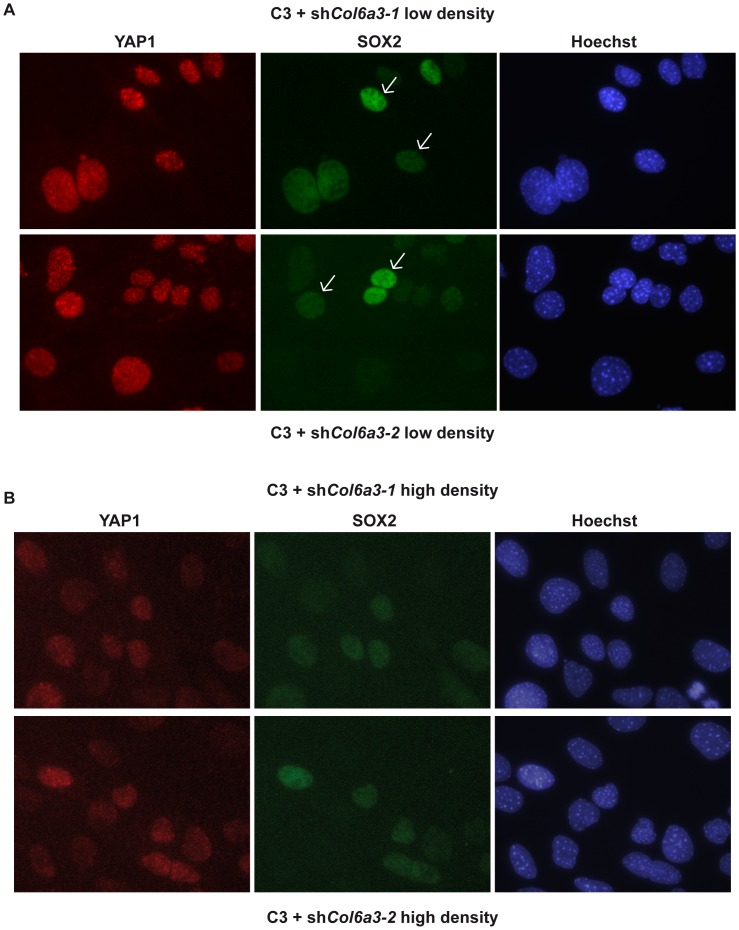
Reactivation of Hippo signalling upon Col6a3 silencing. **A**. Expression of YAP1 and SOX2 in low-density sh*Col6a3* knockdown cells grown for 2 days as monolayers. Cells expressing low or high levels of SOX2 are indicated by arrows. **B**. Expression of YAP1 and SOX2 expression in dense sh*Col6a3* silenced cells grown for 8 days as monolayers. *Col6a3* silencing leads to diminished YAP1 and SOX2 expression.

Importantly, we also observed that *Wnt9a* expression was less strongly induced in dense sh*Col6a3* cells than in normal dense C3 cells, but that *Sfrp2* expression was strongly induced (Fig. 8D). Moreover, we also found that Kibra expression was strongly stimulated in the sh*Col6a3* cells (Fig. 8D). The elevated Kibra expression seen in sh*Col6a3* cells suggests re-activation of Hippo signalling to repress 3D growth. Low density sh*Col6a3* C3 cells show nuclear staining for YAP1 and TAZ and heterogeneous staining for SOX2 analogous to native C3 cells (Fig. 9A and S5A). However, in agreement with the elevated Kibra expression, in dense sh*Col6a3* C3 cells strongly reduced YAP1, TAZ and SOX2 expression is observed (Figs. 9B and S5B). Thus, elevated *Col6a3* expression in dense C3 cells plays a critical role in repressing Hippo signalling to promote 3D growth.

## Discussion

### Cross-talk between Col6a3, TAF4 and the Wnt and Hippo pathways regulates 3D growth

In this study, we show that *Col6a3* expression is up-regulated in densely growing MEFs and that its high expression in *Taf4^−/−^* MEFs promotes loss of contact inhibition and their 3D growth through modulation of the Hippo and Wnt pathways. It is important to note that this phenotype is seen in the two independent *Taf4^−/−^* MEF lines that we isolated and that re-expression of exogenous TAF4 restores cell morphology and promotes contact inhibition. Thus, all of the changes are directly due to loss of TAF4 and can be reversed by its re-expression [4]. *Col6a3* expression is induced in dense TAF4-expressing and *Taf4* ^−/−^ MEFs. The high expression seen in the *Taf4* ^−/−^ MEFs originates mainly from the small number of cells forming foci, whereas in dense TAF4-expressing MEFs, expression is much more homogeneous (data not shown). The ability of TAF4 to counteract the growth promoting effects of high *Col6a3* expression can be evaluated by comparing the responses of the two cell types under conditions of dense growth. In TAF4-expressing C1 cells, YAP1 and TAZ are located in the nucleus as the cells proliferate at low density. At high density, Kibra remains highly expressed, while *Fat4* expression is strongly induced. This suggests that Hippo signalling is maintained in dense C1 cells to mediate contact inhibition, an idea confirmed by the translocation of YAP1 from the nucleus and the overall down-regulation of TAZ expression in dense cultures.

In contrast, in the absence of TAF4, the pathways and factors that normally maintain Kibra expression under dense conditions are no longer operative and Kibra expression is repressed attenuating Hippo signalling. Consequently, in dense *Taf4^−/−^* cells, YAP1 accumulates in the nucleus to promote 3D growth. Kibra expression can be induced by YAP1 overexpression through an as yet unknown mechanism [21]. Nevertheless, the transcription factors and pathways that regulate Kibra expression in the C3 cells and that require TAF4 as a coactivator remain to be determined. It is also interesting to note that FAT4 expression also correlates with that of Hippo signalling despite the fact that current evidence does not support a role for FAT4 in this pathway in mammals where it is rather a critical regulator of the planar cell polarity pathway [30]. The role, if any, that TAF4 control of FAT4 expression may play in modulating the growth of C1 and C3 MEFs remains to be investigated.

Several lines of evidence indicate that Wnt signalling is also involved in 3D growth of *Taf4^−/−^* MEFs. In dense *Taf4^−/−^* MEFs, *Wnt9a* expression is strongly up-regulated while *Sfrp2* expression is repressed. The nuclear localisation of β-catenin in the cells forming 3D foci shows enhanced Wnt signalling in these cells. Also shRNA-mediated *Wnt9a* silencing or use of chemical inhibitors of Wnt signalling abrogates 3D growth indicating the critical role of the pathway in this process. Interestingly, ChIP-seq has revealed SOX2 [31] and YAP1 [26] binding close to the *Wnt9a* gene. This suggests that YAP1 and SOX2 in dense C3 cells may activate *Wnt9a* expression in a positive feed forward loop to promote 3D growth. This contrasts with the TAF4-expressing MEFs, where *Wnt9a* expression is not induced under dense conditions, but *Sfrp2* expression is strongly induced. Consequently, in dense TAF4-expressing MEFs, Wnt signalling is repressed, the opposite of what is observed in *Taf4* ^−/−^ MEFs. The loss of TAF4 therefore modifies *Wnt9a* and *Sfrp* expression to activate Wnt signalling in conditions of high density to promote 3D growth.

Together our results support a model where loss of contact inhibition through diminished Hippo signalling allows the cells to form dense foci, while enhanced Wnt signalling is further required for full 3D growth. It is also interesting to note that high SOX2 expression is seen already in rare nuclei of low-density C3 cells. Thus TAF4 inactivation leads to heterogeneity in the cell population suggesting that it is the SOX2 high population that is competent to generate 3D foci under dense conditions.

### Retinoic acid regulates MEF growth via repression of Col6a3

The capacity of *Taf4* ^−/−^ MEFs for 3D growth appears to be associated with high *Col6a3* expression. The expression of several membrane and ECM components is strongly induced in dense conditions. Nevertheless, shRNA mediated silencing of *Col6a3* alone is sufficient to abolish 3D growth. Further evidence for a critical role of *Col6a3* in 3D growth comes from the observation that its expression is down-regulated by ATRA that restores contact inhibition and represses 3D growth. ATRA down-regulates both *Col6a3* and to a lesser extent *Col6a2*, thereby down-regulating holo-collagen VI fibre formation. While the expression of many membrane and ECM components are strongly induced by 3D growth, *Col6a3* is one of the few regulated by ATRA and is the most strongly repressed. Moreover, ATRA treatment does not affect expression of known components of the Hippo pathway and may even potentiate Wnt signalling through up-regulation of *Wnt9a*. These observations, together with the results of *Col6a3* silencing, indicate that the major mechanism by which ATRA inhibits 3D growth is through repression of *Col6a3*. The growth suppressive effect of ATRA on these cells by regulation of ECM components is therefore fundamentally different from that seen in F9 embryonal carcinoma cells, HL60 myeloid cells, or mammary carcinoma cells where RA treatment induces cell cycle arrest, differentiation and under some conditions apoptosis [28], [29], [32], [33].

ECM components such as COL6A3 can provide a network that physically facilitates 3D growth. While the reduction in this network in the presence of ATRA may contribute to its ability to repress 3D growth, remodelling the ECM does not appear to be the only role of COL6A3 as it's silencing also dramatically modulates gene expression and signalling. Sh*Col6a3* silencing stimulates expression of Kibra and re-activates Hippo signalling leading to reduced YAP1 expression. These observations show that *Col6a3* plays an active role in modulating expression of growth control genes and are in line with previous results showing that high *Col6a3* expression modulates cell and tumour growth. Ovarian cancer cells resistant to cisplatin show a potent induction of the *Col6a3* gene and *in vivo*, high grade tumours express higher levels of *Col6a3* than low grade tumours [13]. Similarly, *Col6a3* is up-regulated in the stroma of colon tumours [34], and promotes the development of hyperplastic foci and primary tumour growth in breast cancer models by activating pro-survival and proliferation pathways involving, as seen here, β-catenin [12]. Collagen VI has also been shown to promote cell cycle progression and anti-apoptotic pathways in serum-starved fibroblasts and in corneal derived fibroblasts [35], [36]. Our data extend these observations showing how *Col6a3* can modulate expression of critical regulators of the Hippo and Wnt pathways to promote growth and how it can serve as a target for ATRA mediated suppression of growth.

Together the results described here reveal a novel and complex interplay between at least three signalling pathways (Hippo, Wnt and ATRA) that control 3D fibroblast growth. We describe the ability of TAF4 to control expression of critical components of the Hippo and Wnt pathways and a novel role of COL6A3 as ATRA-regulated modulator of 3D growth and as regulator of gene expression.

## Supporting Information

Figure S1 **Expression and localisation of TAZ and SOX2 in C1 MEFs.** **A**. Immunostaining of non-dense and dense C1 MEFs for TAZ. **B**. Immunostaining of non-dense and dense C1 MEFs for SOX2 (20× magnification). **C**. Control staining of F9 embryonal carcinoma cells and of hepatocyte cells with SOX2 antibody to demonstrate the specificity of the signal.
(PDF)

Figure S2 **Expression and localisation of TAZ, YAP1 and SOX2 in C3 MEFs.** **A**. Immunostaining of non-dense and dense C3 MEFs for TAZ. **B**. Immunostaining of low density C3 cells with YAP1 and SOX2 antibody (20× magnification). Cells expressing low or high levels of SOX2 are indicated by arrows. **C** Immunostaining of dense C3 MEFs for YAP1 and SOX2 (20× magnification). The location of cells growing in a 3D foci is indicated.
(PDF)

Figure S3 **A. Effect of RA on C3 cell proliferation.** **A**. Kinetics of cell growth in presence or absence of RA as evaluated by cell counting. **B**. Assessment of cell division by incorporation of BrdU on cells grown for the indicated periods in presence or absence of RA. **C**. Results of a representative FACS assay showing the % cells in each stage of cell cycle. D. Clonogenic assays of C3 cells in presence or absence of ATRA or sh*Col6a3* on wells coated with fibronectin.
(PDF)

Figure S4 **Effect of sh** ***Col6a3*** **knockdown on gene expression.** RT-qPCR on the indicated genes in C3 cells expressing control shRNA or shRNA directed against Col6a3 grown for 3 or 10 days as indicated.
(PDF)

Figure S5 **Expression of TAZ in sh** ***Col6a3*** **knockdown cells.** **A**. Expression of TAZ in low-density sh*Col6a3* knockdown cells. **B** Expression of TAZ in high density sh*Col6a3* knockdown cells.
(PDF)

Table S1 **Sequences of primers used for qPCR of the indicated genes on the forward and reverse strands.**
(DOC)

Table S2 **Excel table of RNA-seq results. Page 1 shows transcripts induced in dense conditions.** Shown are, the Ensembl gene ID, the average RPKM expression values under each condition, the fold change and Log2 change values under the indicated conditions, gene name and description. Pages 2–4 show the same information concerning transcripts induced in spheres, repressed under dense conditions and repressed in spheres respectively.
(XLS)

Table S3 **Ontology analyses of genes whose expression is modified under conditions of dense or 3D growth.** Each page shows the analysis of genes differentially regulated under the specified conditions with the indicated ontology terms.
(XLS)

Table S4 **Genes regulated by ARTA in C3 MEFs.** Pages 1 and 2 show the induced and repressed genes after 12 and 72 hours of ATRA treatment. Shown are the Ensembl gene IDs, gene name, log2 ratios −ATRA/+ATRA 12 hours, −ATRA/+ATRA 72 hours, +ATRA12 hours/+ATRA 72 hours, and gene description.
(XLS)
