# Supplementary figures and images for: TAF4 Inactivation Reveals the 3 Dimensional Growth Promoting Activities of Collagen 6A3
Source: PLoS One. 2014 Feb 3;9(2):e87365. doi: 10.1371/journal.pone.0087365 (PMC3911972; doi:10.1371/journal.pone.0087365)

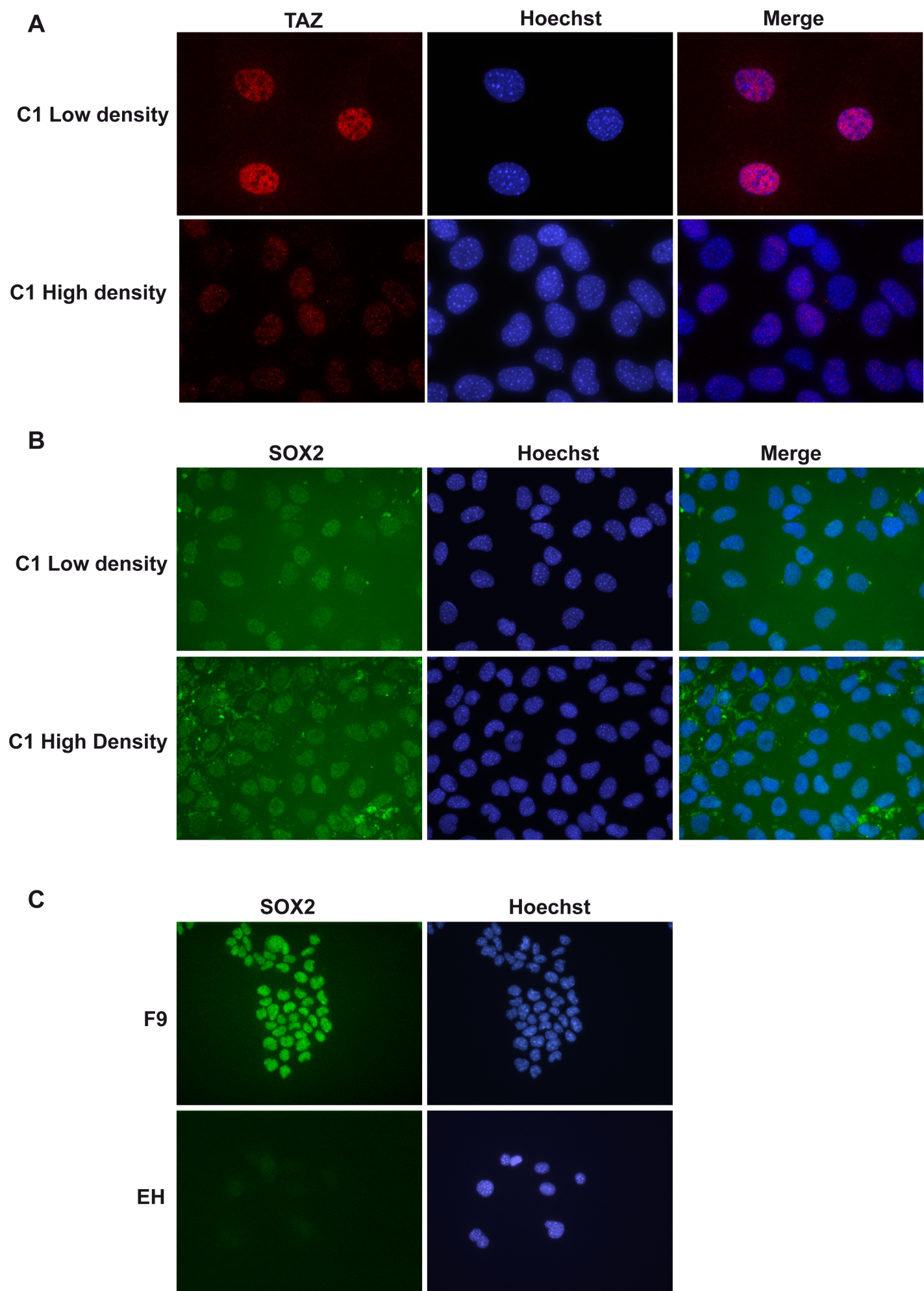

Supplement: Figure S1 — Expression and localisation of TAZ and SOX2 in C1 MEFs. A. Immunostaining of non-dense and dense C1 MEFs for TAZ. B. Immunostaining of non-dense and dense C1 MEFs for SOX2 (20× magnification). C. Control staining of F9 embryonal carcinoma cells and of hepatocyte cells with SOX2 antibody to demonstrate the specificity of the signal. (PDF) [file pone.0087365.s001.pdf]

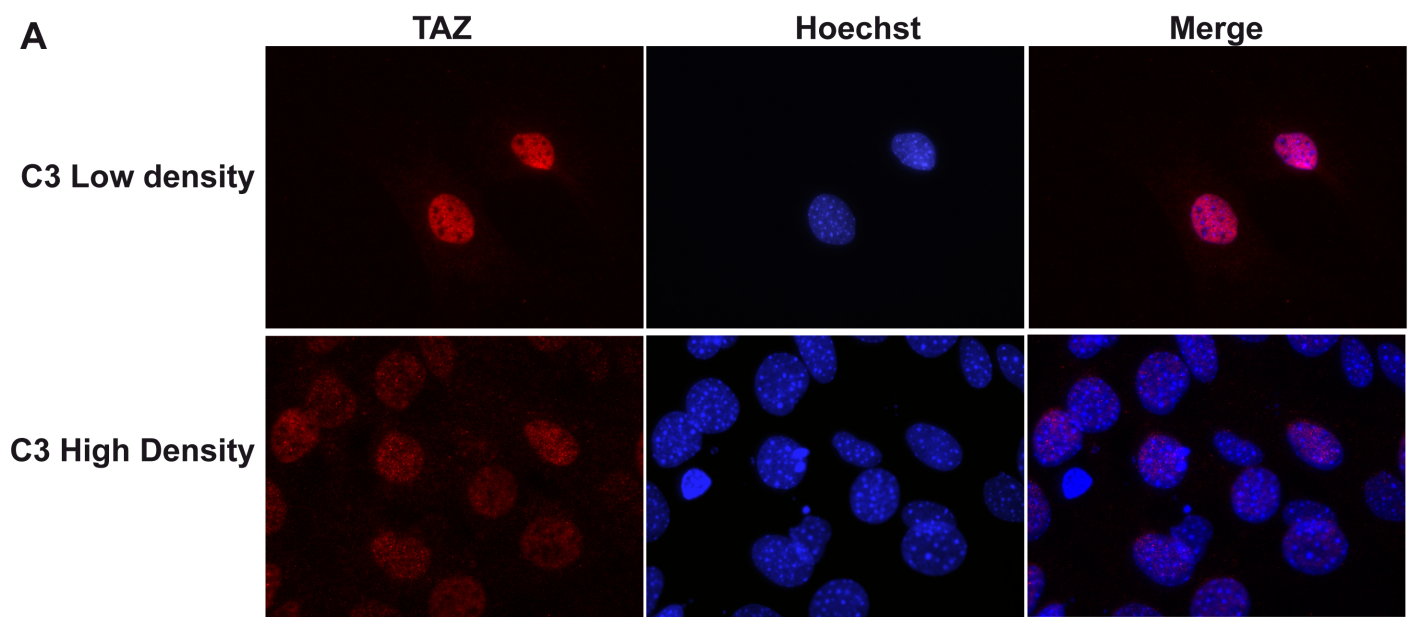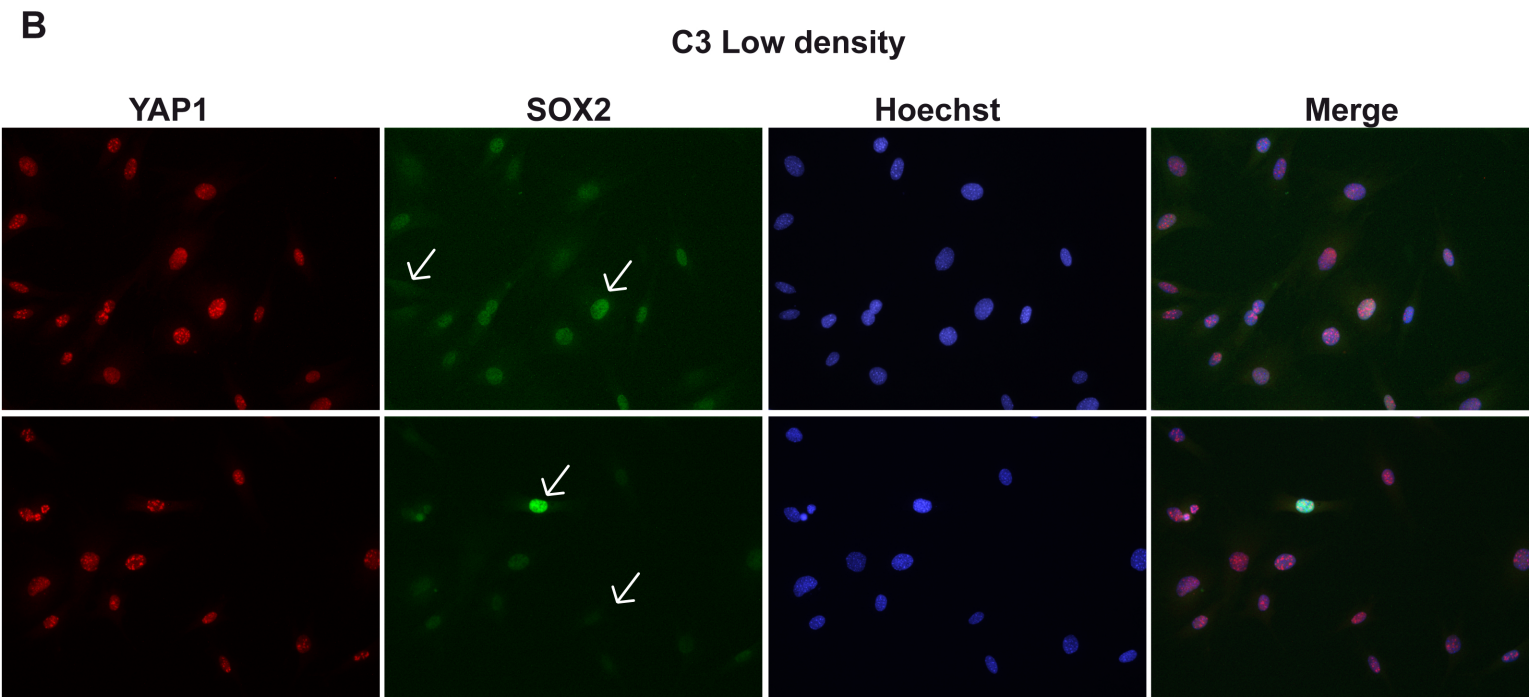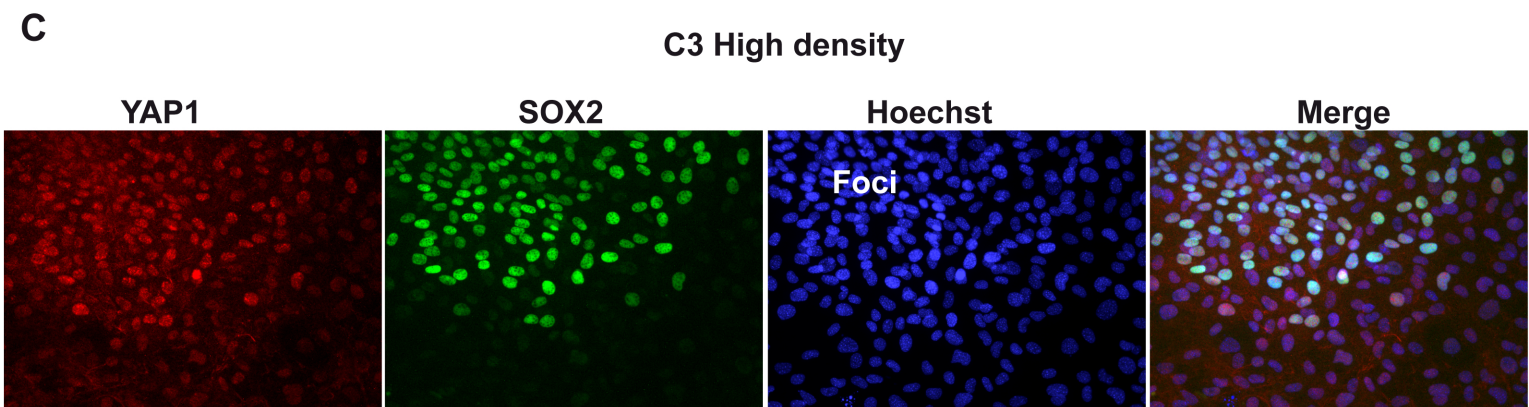

Supplement: Figure S2 — Expression and localisation of TAZ, YAP1 and SOX2 in C3 MEFs. A. Immunostaining of non-dense and dense C3 MEFs for TAZ. B. Immunostaining of low density C3 cells with YAP1 and SOX2 antibody (20× magnification). Cells expressing low or high levels of SOX2 are indicated by arrows. C Immunostaining of dense C3 MEFs for YAP1 and SOX2 (20× magnification). The location of cells growing in a 3D foci is indicated. (PDF) [file pone.0087365.s002.pdf]

**A**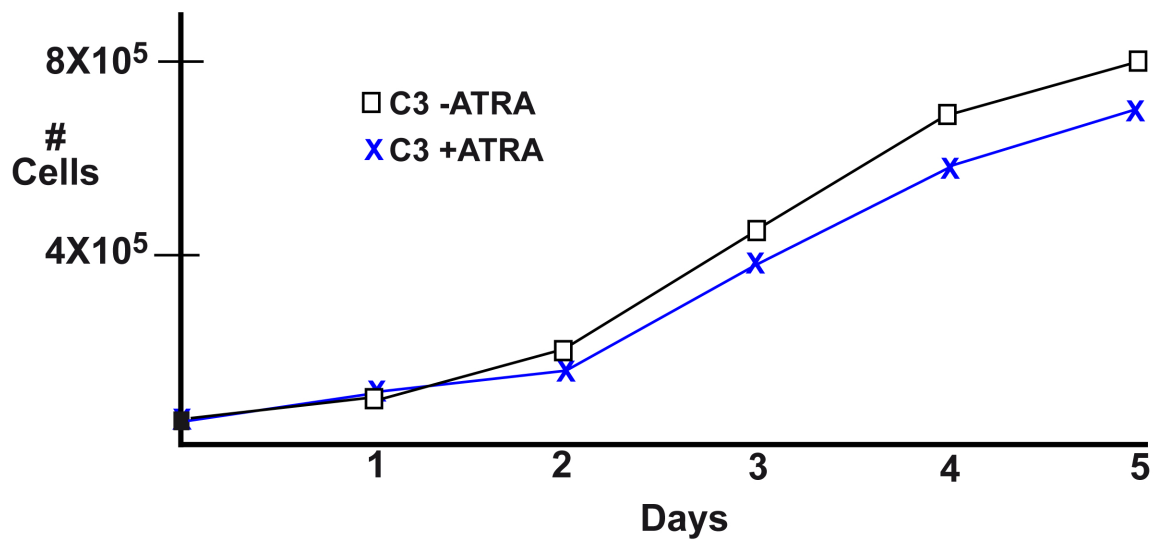**B**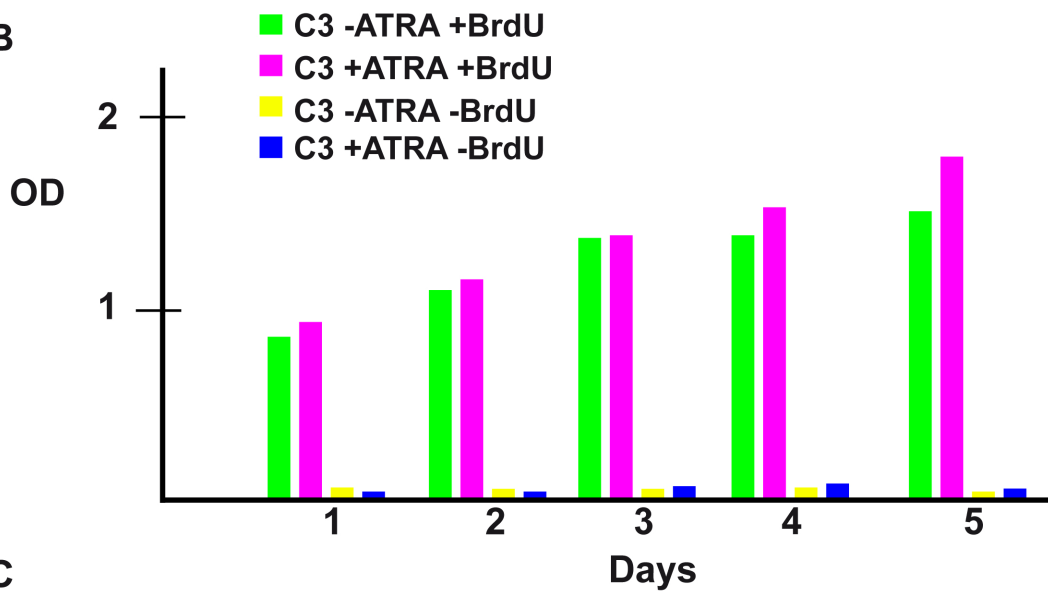**C**

|       |    | G1 | S  | G2 |
|-------|----|----|----|----|
| -ATRA | 24 | 46 | 26 | 26 |
|       | 48 | 59 | 20 | 19 |
| +ATRA | 24 | 49 | 19 | 28 |
|       | 48 | 56 | 23 | 19 |

Supplement: Figure S3 — A. Effect of RA on C3 cell proliferation. A. Kinetics of cell growth in presence or absence of RA as evaluated by cell counting. B. Assessment of cell division by incorporation of BrdU on cells grown for the indicated periods in presence or absence of RA. C. Results of a representative FACS assay showing the % cells in each stage of cell cycle. D. Clonogenic assays of C3 cells in presence or absence of ATRA or shCol6a3 on wells coated with fibronectin. (PDF) [file pone.0087365.s003.pdf]

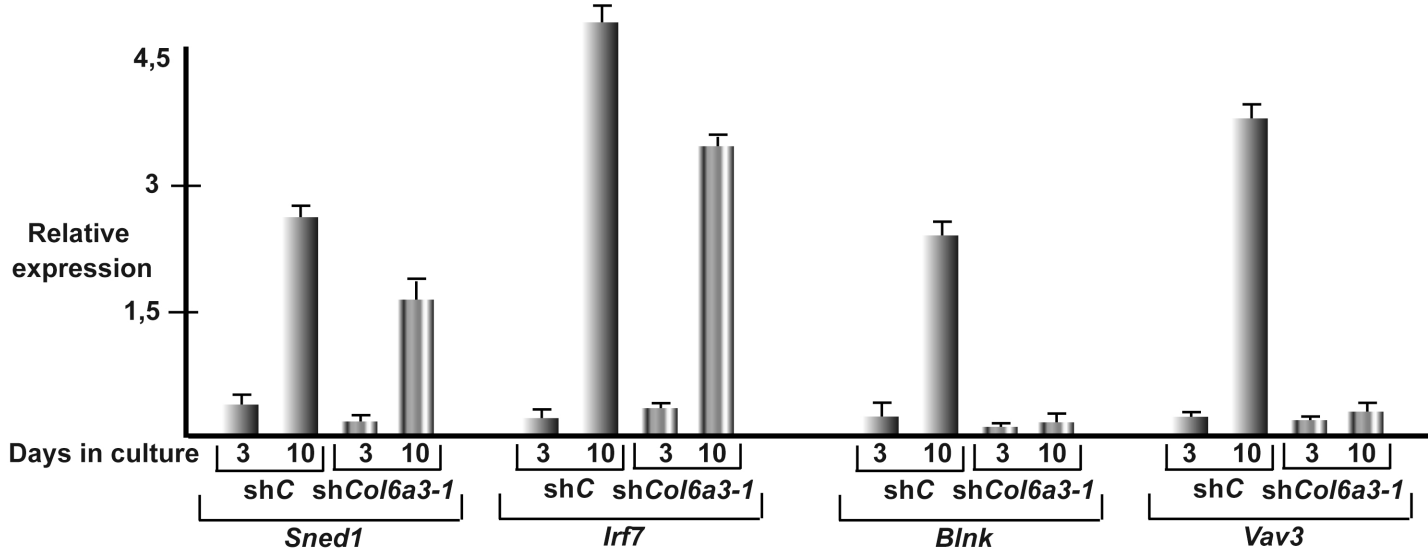

Martianov et al., Figure S4

Supplement: Figure S4 — Effect of sh Col6a3 knockdown on gene expression. RT-qPCR on the indicated genes in C3 cells expressing control shRNA or shRNA directed against Col6a3 grown for 3 or 10 days as indicated. (PDF) [file pone.0087365.s004.pdf]
