# Supplementary material for: TAF4 Inactivation Reveals the 3 Dimensional Growth Promoting Activities of Collagen 6A3
Source: PLoS One. 2014 Feb 3;9(2):e87365. doi: 10.1371/journal.pone.0087365 (PMC3911972; doi:10.1371/journal.pone.0087365)
Supplement: Figure S5 — Expression of TAZ in sh Col6a3 knockdown cells. A. Expression of TAZ in low-density shCol6a3 knockdown cells. B Expression of TAZ in high density shCol6a3 knockdown cells. (PDF) [file pone.0087365.s005.pdf]

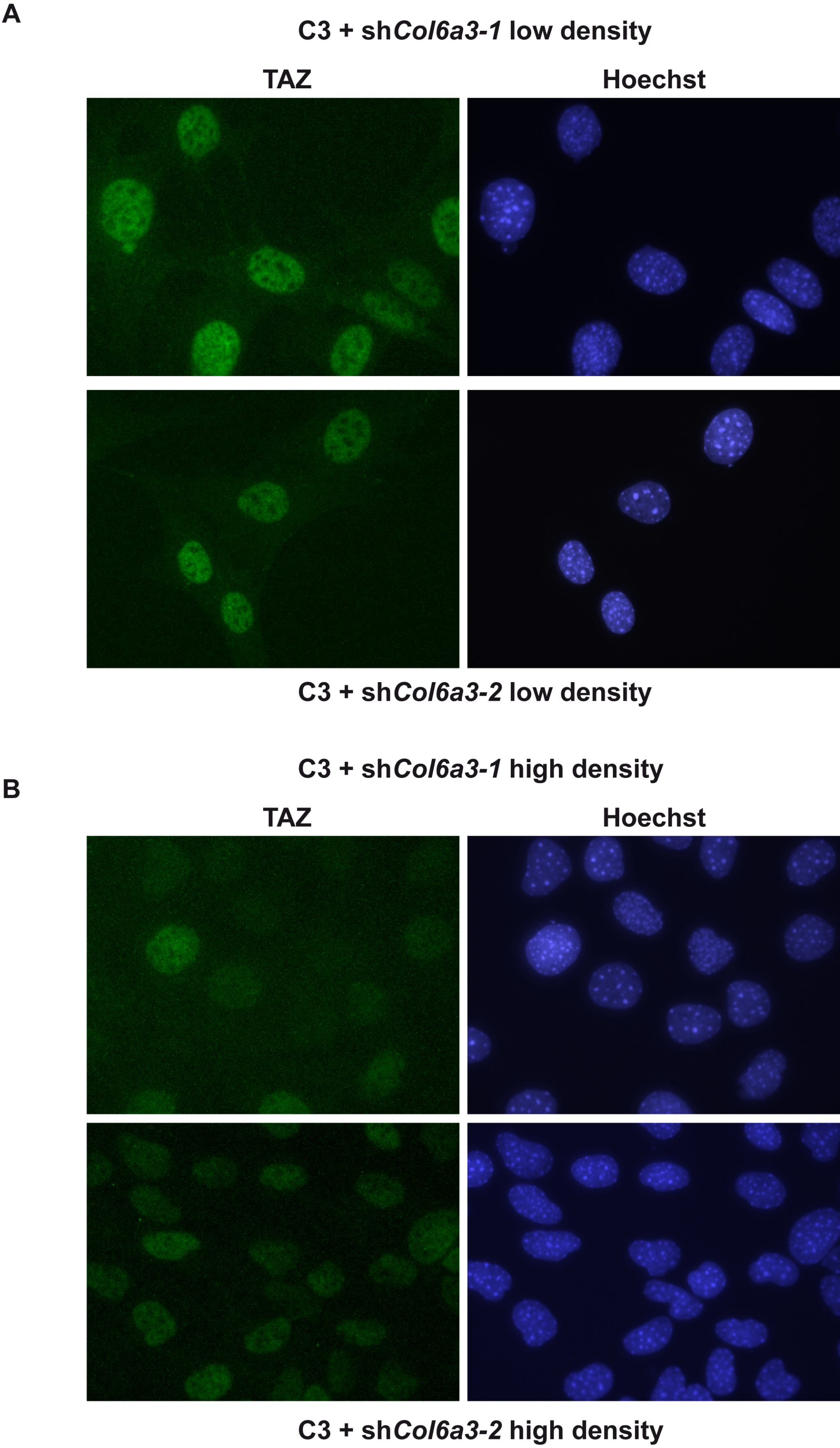

Martianov et al., Figure S5
