# Supplementary material for: TAF4 Inactivation Reveals the 3 Dimensional Growth Promoting Activities of Collagen 6A3
Source: PLoS One. 2014 Feb 3;9(2):e87365. doi: 10.1371/journal.pone.0087365 (PMC3911972; doi:10.1371/journal.pone.0087365)
Supplement: Table S1 — Sequences of primers used for qPCR of the indicated genes on the forward and reverse strands. (DOC) [file pone.0087365.s006.doc]

**Primers for RT qPCR.**

| Name | Sequence (5’to 3’) |
| --- | --- |
| Col6a3 forward  Col6a3 reverse  Sned1 forward  Sned1 reverse  Fgf2 forward  Fgf2 reverse  Mgp forward  Mgp reverse  Vav3 forward  Vav3 reverse  Irf7 forward  Irf7 reverse  Usp18 forward  Usp18 reverse  Olfm1 forward  Olfm1 reverse  Spp1 forward  Spp1 reverse  Wnt9a forward  Wnt9a reverse  Sfrp2 forward  Sfrp2 reverse  Wwc1 forward  Wwc1 reverse  Fat4 forward  Fat4 reverse  Penk1 forward  Penk1 reverse  Ccl5 forward  Ccl5 reverse  Ccl2 forward  Ccl2 reverse  Slp1 forward  Slp1 reverse  Blnk forward  Blnk reverse  Tgm2 forward  Tgm2 reverse  Dhrs3 forward  Dhrs3 reverse  Mme forward  Mme reverse | GAACCACGGAAGAGAGCAAG  CGGCTTCACATCAAGTTCCT  CTTCAGCTCCACTGAACGTG  AGCGGCTCTTGGTACTTTGA  GGCTGCTGGCTTCTAAGTGT  CCGTTTTGGATCCGAGTTTA  ACAGGAGAAATGCCAACACC  GTAGTCATCGCAGGCCTCTC  TCCCAAGTGATGCTGTCAAA  TTTGGTCCTGTGCCTTACAA  GAAGACCCTGATCCTGGTGA  CCAGGTCCATGAGGAAGTGT  CCTGGAAGGATGTCCAGTGT  TTGAAATGCAGCAGACAAGG  AGCTGAGGCAGCTACTGGAG  AACTTGGTCTCCAGGCCTTT  TGACCCATCTCAGAAGCAGA  CTCCATCGTCATCATCATCG  GGAGTGCCAGTACCAGTTCC  GGCAGAAGAGATGGCGTAGA  ACGACAACGACATCATGGAA  ACGCCGTTCAGCTTGTAAAT  TTCCGCTTGCTACTGAGGAT  TGCCATTTTCTCCCTGAAAG  AGCACAAGGCATTCTTGACC  CAAGGCAGTGTCTGGAGTGA  AGCCAGGACTGCGCTAAAT  TGTTATCCCAAGGGAACTCG  CCCTCACCATCATCCTCACT  GAGCACTTGCTGCTGGTGTA  CCCAATGAGTAGGCTGGAGA  TCTGGACCCATTCCTTCTTG  AAGTGCGTGAATCCTGTTCC  TGTATTTGCCGTCACACTGC  AAGTCAAAGGCCCTCCAAGT  CGGAGTCCGAATGTTCATCT  CTAAGAGTGTGGGCCGTGAT  GCCAGTTTGTTCAGGTGGTT  GGTCCATGGAAAAAGCTTGA  ACAATATGGCCGTTCTGGAG  GAAATTCAGCCAAAGCAAGC  TCGGCCTGAGGAATAAAATG |

**shRNA vectors used for *Col6a3* and *Wnt9a* silencing.**

| shRNA | TRC number |
| --- | --- |
| Col6a3-1  Col6a3-2  Wnt9a-1  Wnt9a-2 | TRCN0000091857  TRCN0000091856  TRCN0000071965  TRCN0000071967 |
